# Supplementary figures and images for: The association between microRNA-21 and hypertension-induced cardiac remodeling
Source: PLoS One. 2020 Feb 10;15(2):e0226053. doi: 10.1371/journal.pone.0226053 (PMC7010249; doi:10.1371/journal.pone.0226053)

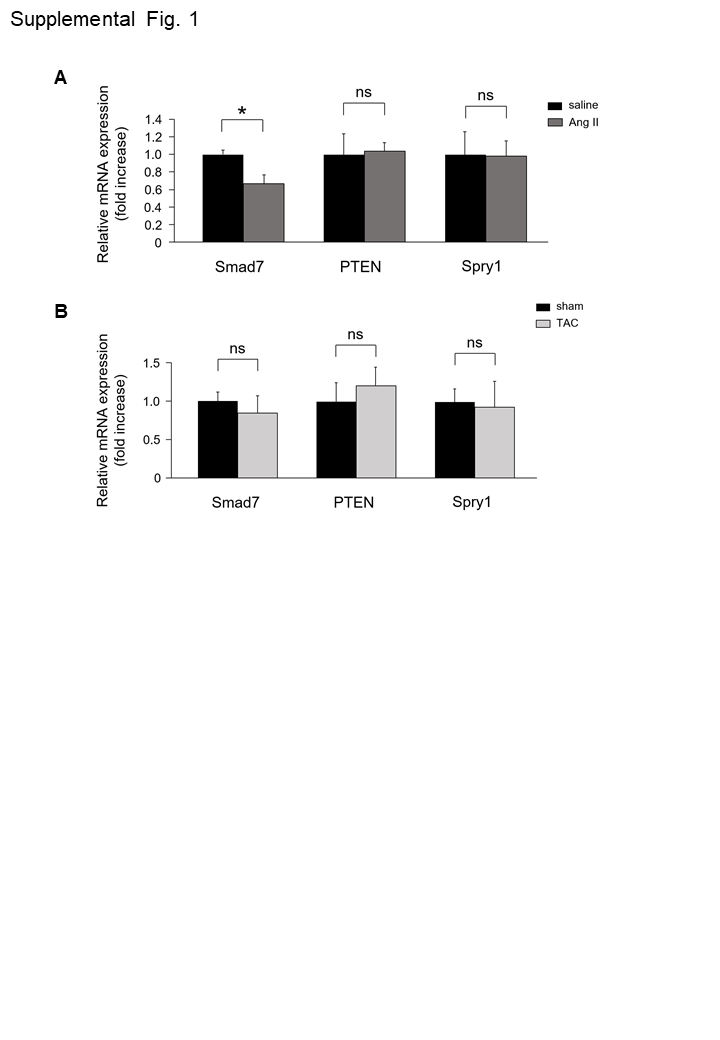

Supplement: S1 Fig — (A) Smad7, PTEN, and Spry1 mRNA levels in Ang II infused mice compared with those of saline infused mice (n = 6 per group). (B) Smad7, PTEN, and Spry1 mRNA levels in TAC mice compared with those of sham mice (n = 6 per group). Data are expressed as mean ± SEM. *P < 0.05. (TIF) [file pone.0226053.s001.TIF]

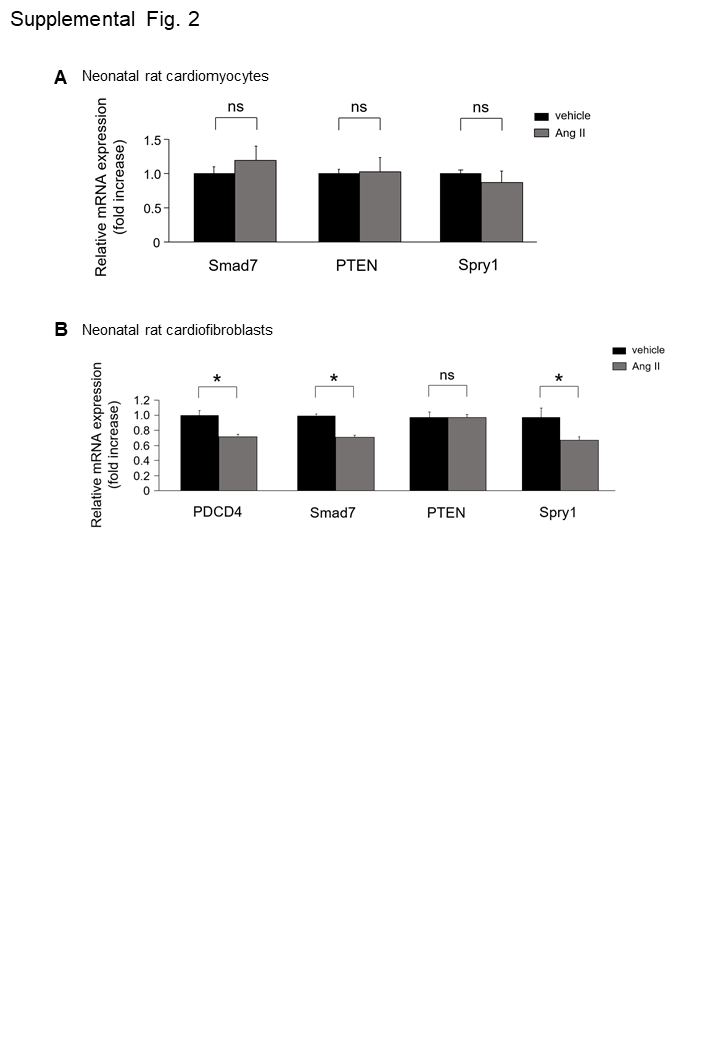

Supplement: S2 Fig — (A) The mRNA expressions of Smad7, PTEN, and Spry1 after treatment with vehicle or Ang II for 24 h in NRCMs (n = 4–6 per group). (B) The mRNA expressions of PDCD4, Smad7, PTEN, and Spry1 after treatment with vehicle or Ang II for 24 h in cardiofibroblasts (n = 4–6 per group). Data are expressed as mean ± SEM. *P < 0.05. (TIF) [file pone.0226053.s002.TIF]
