# Supplementary material for: The association between microRNA-21 and hypertension-induced cardiac remodeling
Source: PLoS One. 2020 Feb 10;15(2):e0226053. doi: 10.1371/journal.pone.0226053 (PMC7010249; doi:10.1371/journal.pone.0226053)
Supplement: S1 Raw images — (PPTX) [file pone.0226053.s003.pptx]

## Slide 1
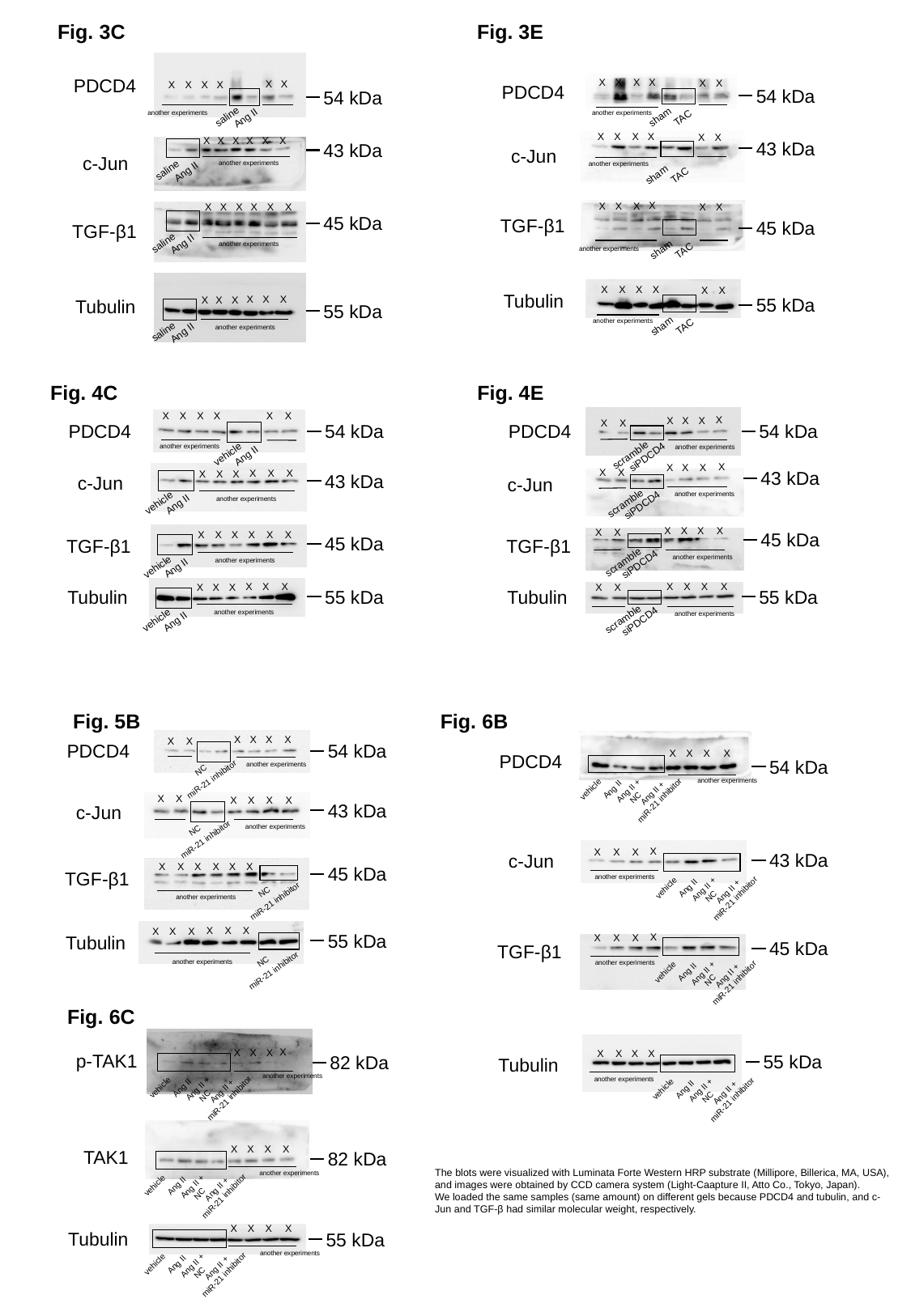

Fig. 3C
Fig. 3E
PDCD4
X
X
X
X
X
X
X
X
X
X
X
X
PDCD4
54 kDa
54 kDa
another experiments
another experiments
saline
sham
TAC
Ang II
X
X
X
X
X
X
X
X
X
X
X
X
43 kDa
43 kDa
c-Jun
c-Jun
another experiments
another experiments
saline
Ang II
sham
TAC
X
X
X
X
X
X
X
X
X
X
X
X
45 kDa
TGF-β1
45 kDa
TGF-β1
saline
Ang II
another experiments
sham
TAC
another experiments
X
X
X
X
X
X
Tubulin
X
X
X
X
X
X
55 kDa
Tubulin
55 kDa
another experiments
sham
TAC
another experiments
saline
Ang II
Fig. 4C
Fig. 4E
X
X
X
X
X
X
X
X
X
X
X
X
54 kDa
54 kDa
PDCD4
PDCD4
another experiments
another experiments
vehicle
scramble
Ang II
siPDCD4
X
X
X
X
X
X
43 kDa
X
X
X
X
X
X
43 kDa
c-Jun
c-Jun
another experiments
another experiments
vehicle
scramble
Ang II
siPDCD4
X
X
X
X
X
X
45 kDa
X
X
X
X
X
X
45 kDa
TGF-β1
TGF-β1
another experiments
scramble
another experiments
siPDCD4
vehicle
Ang II
X
X
X
X
X
X
X
X
X
X
X
X
55 kDa
55 kDa
Tubulin
Tubulin
another experiments
another experiments
scramble
vehicle
siPDCD4
Ang II
Fig. 6B
Fig. 5B
X
X
X
X
X
X
PDCD4
54 kDa
X
X
X
X
PDCD4
54 kDa
another experiments
NC
miR-21 inhibitor
another experiments
Ang II +
NC
vehicle
Ang II
Ang II +
miR-21 inhibitor
X
X
X
X
X
X
43 kDa
c-Jun
another experiments
NC
miR-21 inhibitor
X
X
X
X
43 kDa
c-Jun
X
X
X
X
X
X
45 kDa
TGF-β1
another experiments
Ang II +
NC
vehicle
Ang II
Ang II +
miR-21 inhibitor
NC
another experiments
miR-21 inhibitor
X
X
X
X
X
X
55 kDa
X
X
X
X
Tubulin
45 kDa
TGF-β1
NC
another experiments
another experiments
miR-21 inhibitor
Ang II +
NC
vehicle
Ang II
Ang II +
miR-21 inhibitor
Fig. 6C
X
X
X
X
X
X
X
X
p-TAK1
55 kDa
82 kDa
Tubulin
another experiments
another experiments
Ang II +
NC
vehicle
Ang II
Ang II +
NC
vehicle
Ang II
Ang II +
miR-21 inhibitor
Ang II +
miR-21 inhibitor
X
X
X
X
TAK1
82 kDa
The blots were visualized with Luminata Forte Western HRP substrate (Millipore, Billerica, MA, USA), and images were obtained by CCD camera system (Light-Caapture II, Atto Co., Tokyo, Japan).
We loaded the same samples (same amount) on different gels because PDCD4 and tubulin, and c-Jun and TGF-β had similar molecular weight, respectively.
another experiments
Ang II +
NC
vehicle
Ang II
Ang II +
miR-21 inhibitor
X
X
X
X
Tubulin
55 kDa
another experiments
Ang II +
NC
vehicle
Ang II
Ang II +
miR-21 inhibitor
